# Health care practitioners’ views of the support women, partners, and the couple relationship require for birth trauma: current practice and potential improvements

**DOI:** 10.1017/S1463423620000407

**Published:** 2020-10-02

**Authors:** Amy Delicate, Susan Ayers, Sarah McMullen

**Affiliations:** 1 PhD Candidate, Centre for Maternal and Child Health Research, School of Health Sciences, City, University of London, London, EC1V 0HB, UK; 2 Professor, Centre for Maternal and Child Health Research, School of Health Sciences, City, University of London, London, EC1V 0HB, UK; 3 Director of Impact and Engagement, NCT, 30 Euston Square, London, NW1 2FB, UK

**Keywords:** barrier, birth trauma, health care practitioner, parent, relationship, support

## Abstract

**Aim:**

To examine health care practitioners’ views of the support women, partners, and the couple relationship require when affected by birth trauma, barriers to gaining such support, and potential improvements.

**Background:**

Ongoing distress following psychologically traumatic childbirth, also known as birth trauma, can affect women, partners, and the couple relationship. Birth trauma can lead to post traumatic stress symptoms (PTSS) or disorder (PTSD). Whilst there is a clear system of care for a PTSD diagnosis, support for the more prevalent experience of birth trauma is not well-defined.

**Method:**

An online survey of health care practitioners’ views of the support parents require for birth trauma, barriers to accessing support, and potential improvements. Practitioners were recruited in 2018 and the sample for the results presented in the article ranged from 95 to 110.

**Results:**

Practitioners reported differing needs of support for women, partners, and the couple as a unit. There was correlation between practitioners reporting having the skills and knowledge to support couples and feeling confident in giving support. The support most commonly offered by practitioners to reduce the impact on the couple relationship was listening to the couple. However practitioners perceived the most effective support was referral to a debriefing service. Practitioners observed several barriers to both providing support and parents accessing support, and improvements to birth trauma support were suggested.

**Conclusions:**

Practitioners indicate that some women, partners, and the couple as a unit require support with birth trauma and that barriers exist to accessing effective support. The support that is currently provided often conflicts with practitioners’ perception of what is most effective. Practitioners indicate a need to improve the identification of parents who need support with birth trauma, and more suitable services to support them.

## Introduction

If a woman or her partner perceives birth as traumatic, it can result in ongoing emotional distress, often referred to as birth trauma. Whilst the term birth trauma lacks universal acceptance or standard definition (Elmir *et al.*, 2010) it is widely used, and recognised that it can lead to post-traumatic stress symptoms (PTSS) or disorder (PTSD) (White *et al.*, 2006; Yildiz *et al.*, 2017). The prevalence of birth trauma is difficult to establish as this term is often used interchangeably with PTSS and PTSD. However, evidence suggests approximately 20%–40% of women report their birth as traumatic (Creedy *et al.*, 2000; Soet *et al.*, 2003; Alcorn *et al.*, 2010; Ayers *et al.*, 2018), with health care practitioners perceiving 30% of women and a quarter of partners to be affected by birth trauma (Delicate *et al.*, 2020). Systematic reviews find that up to 16.8% of women experience clinically significant post-traumatic stress symptoms such as: re-experiencing, hyperarousal, avoidance, and negative alterations to cognition (Dekel *et al.*, 2017) and post-partum PTSD affects 3% to 4% of women overall (Yildiz *et al.*, 2017). There is also evidence to suggest that birth trauma can effect partners causing them ongoing distress (Etheridge and Slade, 2017), depression (Hinton *et al.*, 2014), and PTSS (Bradley *et al.*, 2008).

Birth trauma and PTSD are highly comorbid with anxiety (Dikmen-Yildiz *et al.*, 2017), depression (Ayers *et al.*, 2016), and secondary tokophobia (Hofberg and Brockington, 2000). For mothers, post-partum PTSD is associated with not initiating breastfeeding (Garthus-Niegel *et al.*, 2018a) and perceived reduced bond with their baby (Dekel *et al.*, 2018). There is evidence to suggest that birth trauma can have a negative impact on couple relationships such as strain on the relationship and loss of intimacy (Delicate *et al.*, 2018a). Likewise, post-partum PTSS is associated with experiencing depression symptoms, which are related to poor relationship satisfaction (Garthus-Niegel *et al.*, 2018b). Whilst reduction of couple relationship satisfaction is a common observation in the transition to parenthood, the impact of birth trauma appears to be more negative (Delicate *et al.*, 2018b). The quality of the couple relationship is an important issue as it is associated with individual well-being and child outcomes (Cowan and Cowan, 2019).

Parents affected by birth trauma may require support to address trauma symptoms and overcome any impact these have on their life. It is important that such support addresses the needs of women, partners, and the relationships between them and their infant (NHS England, 2015). This support may come from health care practitioners such as midwives, health visitors (public health nurse), physicians/general practitioners (GP), or specialist mental health care practitioners (Ayers and Shakespeare, 2015). In addition, parents may also access support from third sector (non-profit and non-government) organisations and engage with non-clinical services such as parent education (Ayers and Delicate, 2016).

Whilst awareness of the importance of perinatal mental health care is rising, clinical guidelines seldom refer to birth trauma. An exception is seen in the UK which has a public funded national health service (NHS) guided by the National Institute for Health and Care Excellence (NICE). NICE recommend that practitioners offer advice and support to women who report birth as traumatic and consider that their partner may also be affected and require help (NICE, 2020). Where post-partum PTSD is identified, a stepped system of care is recommended of: (1) watchful waiting and (2) trauma-focussed cognitive behaviour therapy or eye movement desensitisation and reprocessing (NICE, 2018a).

When tailored to meet the needs of post-partum women, trauma-focussed psychological therapies have been shown to be effective in reducing PTSD symptoms in the early post-partum period (Furuta *et al.*, 2018). Midwife-led debriefing, whilst routinely provided for birth trauma (Ayers *et al.*, 2006) has no robust evidence for effectiveness (Bastos *et al.*, 2015; de Graaff *et al.*, 2018). However, women appear to value debriefing services, also known as birth listening and reflections, particularly women with high levels of PTSS (Meades *et al.*, 2011; Baxter, 2019). Further birth trauma support strategies reported in the literature include: expressive writing (Di Blasio *et al.*, 2015); sharing of birth stories online (Blainey and Slade, 2015); social support (Vesel and Nickasch, 2015); art therapy (Hogan, 2015); hypnosis (Slater, 2015); and information from websites, books and helplines (Thomson *et al.*, 2017).

Women and partners indicate that they want support for birth trauma (Ayers *et al.*, 2006; Etheridge and Slade, 2017), but multi-level barriers exist to accessing perinatal mental health services (Smith *et al.*, 2019). On an individual level, parents can lack awareness of perinatal mental health symptoms (Russell *et al.*, 2017; Das and Hodkinson, 2019), stigma can exist around disclosing mental health concerns (NCT, 2017), and guilt at being emotionally unwell at a perceived time of happiness (Smith *et al.*, 2019). Partners may be particularly reluctant to seek help for their own perinatal mental health (Darwin *et al.*, 2017), and can feel unjustified talking about birth trauma when they witnessed the trauma as oppose to women who go through the physical process of birth (Etheridge and Slade, 2017).

Barriers to health care practitioners supporting parents with birth trauma can exist through lack of knowledge (de Vries *et al.*, 2018) and limited competence in supporting perinatal mental health (Borglin *et al.*, 2015), which could stem from limitations in perinatal mental health training (NHS England, 2015). The quality of inter-personal relationships between parents and health care practitioners can create obstacles to identifying parents with mental health problems (NCT, 2017). Barriers can be created due to lack of continuity of care (Bayrampour *et al.*, 2018), parents having limited time with health care practitioners, and maternal perception that post-partum health care is focussed on infant health (Byrne *et al.*, 2017).

The organisation of health care can present further problems for accessing services, not least deficits in funding for perinatal mental health services (Bauer *et al.*, 2014) and birth trauma specifically (Thomson and Garrett, 2019). Perinatal mental health services are prone to focus on the treatment of depression (Higgins *et al.*, 2018) and moderate to severe disorders in mothers (Russell *et al.*, 2017). The well-being of partners continues to be overlooked by perinatal services with a general lack of tailored support (Baldwin *et al.*, 2018). Research suggests that perinatal mental health outcomes could be improved by consistent, routine assessment with reliable referral pathways, and specialist support services (Noonan *et al.*, 2017; Viveiros and Darling, 2019).

Awareness of perinatal mental health problems is increasing (Ayers and Shakespeare, 2015), and recommendations exist for the identification and support of mental health problems in women and partners (Oates, 2015; NICE, 2020). To date, birth trauma research has mainly focussed on the effect upon and treatment of birthing women, and has largely disregared the needs of partners and the couple as a unit. A recent study of UK health care practitioner found that over one third of women and one quarter of partners are perceived to be affected by birth trauma; with up to 30% of couple relationships being impacted (Delicate *et al.*, 2020). This study also explored the role of support for those affected by birth trauma and these findings are presented in the current article.

The results reported in the present article, aimed to explore the current birth trauma support offered to women and partners as individuals, and a couple, along with practitioners’ views of the most effective support for reducing the impact of birth trauma on the couple relationship. Furthermore, the study aimed to investigate practitioners’ perception of: barriers to couples accessing support; barriers to practitioners giving couples support; and potential improvements in support for couples affected by birth trauma.

## Method

The results presented in this article are taken from a wider study which has already reported on health care practitioners perceptions of the observed rates and impact of birth trauma (Delicate *et al.*, 2020). The study employed an online survey of health care practitioners to aid ease of participation and assist sample size and range of practitioner roles represented in the sample (Wright, 2005).

### Participants

Practitioners were eligible to participate if they were currently working in clinical or non-clinical roles within the UK, supporting parents in the first-year post-partum. A self-selecting convenience sample of UK health care practitioners was utilised, and sample size was not predetermined as the survey was exploratory.

### Procedures

A survey was designed by the authors to address the research aims. The survey questions and response criteria were drawn from existing literature and the design process utilised a small trial with practitioners to refine questions and answer categories. At the beginning of the survey, birth trauma was defined as having experienced ‘emotionally traumatic childbirth causing ongoing distress’ and participants were instructed to respond to the survey based on their experience of working with parents in the first-year post-partum. Ethical approval for the study was obtained from the School of Health Sciences at City, University of London.

Participants were recruited between May and September 2018 through social media (Facebook and Twitter) and organisations such as the Institute of Health Visiting; NCT; Midwives Information and Resources Service (MIDIRS); and the Royal College of General Practitioners. Recruitment information enabled practitioners to link to and download the participant information sheet. Participation was voluntary and anonymous and to participate, practitioners were required to give consent at the start of the survey hosted by Qualtrics. Contact information in the form of an email was only collected if the participant indicated they wished to receive a summary of the study findings. Data were collected and stored within the web-based survey tool Qualtrics and exported to the statistics software SPSS 25 for analysis.

### Analyses

Geographical data was checked and any participants from outside the UK were removed from the sample. The sample was examined using frequencies for practitioner role and length of qualification. Frequencies were calculated for statements about perceived support needs for women, partners, and couples plus practitioner knowledge, skills, and confidence with birth trauma support. Kendall’s tau-b correlations were used to assess relationships between responses to parents’ support needs and practitioner aptitude.

Frequencies were calculated for the support practitioners currently offered to couples and which support practitioners viewed as most effective for reducing the impact on the couple relationship. Fisher’s exact tests were used to examine differences between the support offered to couples, and practitioner’s perception of its effectiveness. The frequencies with which practitioners signposted women, partners, and couples to other organisations were calculated. Practitioner’s views of the barriers to couples accessing support, and practitioners providing couples with support were examined using frequencies.

Open question responses for practitioners’ views on gaps in support services for couples affected by birth trauma, what would help couples access suitable support, and enable practitioners to support couples affected by birth trauma, were analysed using an approach similar to content analysis. For each question, participant responses were coded, and related codes grouped into categories. Due to apparent similarities, categories were compared between questions and rationalised to form over-arching themes for the open question data collectively.

## Results

A sample of 202 health care practitioners engaged in the study and their characteristics are given in prior publication of study findings (Delicate *et al.*, 2020). However, due to participant attrition as the survey progressed, the sample for results reported in this article is 110, and sample characteristics detailed in Table 1.

**Table 1.** Article sample (*n* = 110)

Practitioner Role	*n* (%)
Doula	13 (11.8)
Family Support Worker (Social Care)	2 (1.8)
General Practitioner (Physician)	3 (2.7)
Health Visitor (Public Health Nurse)	29 (26.4)
Breastfeeding Counsellor	1 (0.9)
Maternity Care Assistant	2 (1.8)
Midwife	31 (28.2)
Parent Educator	24 (21.8)
Psychological Counsellor/Therapist	5 (4.5)

Length of Qualification	*n* (%)
Student	9 (8.2)
0–4 Years	27 (24.5)
5–9 Years	30 (27.3)
10–14 Years	20 (18.2)
15–19 Years	10 (9.1)
20+ Years	14 (12.7)

### Parent support needs and practitioner aptitude

Practitioners reported differing levels of need for support from women, partners, and couples, which are detailed in Table 2 along with practitioners’ perception of their own aptitude for giving birth trauma support. Practitioners reported that 54.5% (*n* = 60) of *‘women want me to support them’* with birth trauma *‘a lot’* or *‘a great deal’*. Compared to 6.5% (*n* = 7) of *‘partners want me to support them’* and 19.1% (*n* = 21) of *‘couples want me to support them’* with birth trauma *‘a lot’* or *‘a great deal’*. A strong positive correlation was found between practitioner responses to *‘I have the skills and knowledge required to support couples’* and *‘I feel confident in supporting couples’* (τ_b_ = 0.80, *p* ≤ 0.00). Moderate positive correlations were found between practitioner responses to *‘I have the resources to offer support to couples’ and ‘I feel confident in supporting couples’* (τ_b_ = 0.54, *p* ≤ 0.00), and *‘I have the resources to offer support to couples’* and *‘I have the skills and knowledge required to support couples’* (τ_b_ = 0.60, *p* ≤ 0.00).

**Table 2.** Parent support needs and practitioner aptitude

	Not at all	A little	A moderate amount	A lot	A great deal
Statement	*n* (%)	*n* (%)	*n* (%)	*n* (%)	*n* (%)
Women want me to support them (*n* = 110)	2 (1.8)	18 (16.4)	30 (27.3)	44 (40.0)	16 (14.5)
Partners want me to support them (*n* = 107)	22 (20.6)	60 (56.1)	18 (16.8)	6 (5.6)	1 (0.9)
Couples want me to support them (*n* = 110)	41 (37.3)	46 (41.8)	11 (10.0)	10 (9.1)	2 (1.8)
I have the resources to offer support to couples (*n* = 109)	21 (19.3)	40 (36.7)	30 (27.5)	13 (11.9)	5 (4.6)
I have the skills and knowledge required to support couples (*n* = 110)	10 (9.1)	41 (37.3)	31 (28.2)	22 (20.0)	6 (5.5)
I feel confident in supporting couples (*n* = 109)	9 (8.3)	35 (32.1)	36 (33.0)	21 (19.3)	8 (7.3)
I feel that supporting couples is part of my role (*n* = 110)	6 (5.5)	20 (18.2)	20 (18.2)	31 (28.2)	33 (30.0)

### Birth trauma support

Data for the birth trauma support offered by health care practitioners is detailed in Table 3. The most offered support to couples experiencing birth trauma was listening to the couple (94.5%, *n* = 104), then referral to a birth listening/debriefing service (72.7%, *n* = 80), then self-help for example books and websites (53.6%, *n* = 59). Conversely, the support practitioners felt was most effective in reducing the impact of birth trauma on the couple relationship was birth listening/debriefing service (73.6%, *n* = 81), then listening to the couple (72.7%, *n* = 80), then referral to a mother and baby unit (46.4%, *n* = 51). There were significant differences between practitioner’s rate of offering support and rating it most effective support in reducing the impact on a couple’s relationship for: watchful waiting; listening to the couple; self-help; and charitable/third sector organisations (Fishers Exact Test, *p* < 0.001).

**Table 3.** Current birth trauma support (*n* = 110)

	Offered to Couples Affected by Birth Trauma	Viewed Most Effective in Reducing Impact on Couple Relationship	Difference Fisher’s Exact Test
	*n* (%)	*n* (%)	*P*
Watchful Waiting	42 (38.2)	14 (12.7)	<0.001*
Listening to the couple	104 (94.5)	80 (72.7)	<0.001*
Self Help for example Books and Websites	59 (53.6)	23 (20.9)	<0.001*
Charitable/third-sector organisation	54 (49.1)	29 (26.4)	<0.001*
Birth listening/Debriefing service	80 (72.7)	81 (73.6)	0.337
Individual Psychotherapy	12 (10.9)	33 (30.0)	0.178
Couple Psychotherapy	7 (6.4)	30 (27.3)	1.00
Couple Relationship Therapy	8 (7.3)	33 (30.0)	0.051
Mother and Baby Unit	56 (50.9)	51 (46.4)	0.712

* Significant.

When indicating which type of birth trauma support they felt was most effective, two thirds of practitioners (66.4%, *n* = 73) selected three support types, over a quarter (29.1%, *n* = 32) selected five or more choices. Of the practitioners (*n* = 22) that indicated *‘other’* as a form of support, 31.8% (*n* = 7) stated that rewind therapy was offered, six of those seven practitioners indicating they thought rewind therapy was most effective in reducing the impact on the couple relationship.

Referral to other organisations, presented in Table 4, showed some similarities for third sector services and resources women, partners, and couples were informed about. The Birth Trauma Association (women 44.2%, *n* = 42; partner 33.7%, *n* = 32; couple 36.8%, *n* = 35), PANDA (women 32.6%, *n* = 31; partner 21.1%, *n* = 20; couple 20.0%, *n* = 19), Children Centre (women 30.5%, *n* = 29; partner 14.7%, *n* = 14; couple 20.0%, *n* = 19), Relate (women 29.5%, *n* = 28; partner 24.2%, *n* = 23; couple 46.3%, *n* = 44); and MIND (women 27.4%, *n* = 26; partner 24.2%, *n* = 23; couple 23.2%, *n* = 22). In open text responses to *‘other’* referrals were: local perinatal mental health charities (*n* = 12); birth afterthoughts (*n* = 4); rewind therapy (*n* = 3); Fatherhood Institute (*n* = 1); emotional freedom therapy (*n* = 1); and the GP (*n* = 1).

**Table 4.** Signposting to organisations (*n* = 95)

	Signpost Women	Signpost Partners	Signpost Couples
Organisation	*n* (%)	*n* (%)	*n* (%)
Big White Wall	1 (1.1)	1 (1.1)	2 (2.1)
Birth Trauma Association	42 (44.2)	32 (33.7)	35 (36.8)
Birth Trauma Chat	9 (9.5)	4 (4.2)	7 (7.4)
Children Centre	29 (30.5)	14 (14.7)	19 (20.0)
Family Action	3 (3.2)	2 (2.1)	3 (3.2)
Homestart	18 (18.9)	5 (5.3)	12 (12.6)
MIND	26 (27.4)	23 (24.2)	22 (23.2)
NCT	16 (16.8)	11 (11.6)	15 (15.8)
OnePlusOne	10 (10.5)	9 (9.5)	7 (7.4)
PANDA	31 (32.6)	20 (21.1)	19 (20.0)
Parent Infant Partnership	8 (8.4)	8 (8.4)	7 (7.4)
Relate	28 (29.5)	23 (24.2)	44 (46.3)
Tavistock Relationship	6 (6.3)	6 (6.3)	10 (10.5)
The Relationship Foundation	4 (4.2)	5 (5.3)	5 (5.3)

### Barriers to birth trauma support

Table 5 details practitioners views of the barriers that exist to couples receiving support and practitioners providing support. The three most commonly reported barriers to couples accessing support services that practitioners reported to see *‘a lot’* or *‘a great deal’* were: *‘long waiting times for support services’* (68.6%, *n* = 72); *‘stigma around disclosing problems with couple relationships*’ (67.3%, *n* = 70); and *‘lack of suitable services’* (61.3%, *n* = 65). The three most commonly reported barriers to practitioners providing support to couples that practitioners reported experiencing *‘a lot’* or *‘a great deal’* were: *‘absence of suitable service to refer couple to’* (52.5%, *n* = 52); *‘lack of time to spend personally supporting parents*’ (48.5%, *n* = 48); and *‘lack of contact with partners to identify those with birth trauma*’ (45.8%, *n* = 44).

**Table 5.** Barriers to obtaining birth trauma support

	Not at all	A little	A moderate amount	A lot	A great deal
	*n* (%)	*n* (%)	*n* (%)	*n* (%)	*n* (%)
Barrier to couples accessing support services					
Concerns that requesting support will imply poor parenting (*n* = 105)	15 (14.3)	34 (32.4)	27 (25.7)	16 (15.2)	13 (12.4)
Lack of awareness of childbirth related post-traumatic stress (*n* = 102)	0 (0.0)	21 (20.6)	31 (30.4)	33 (32.4)	17 (16.7)
Lack of childcare provision to access support service (*n* = 104)	11 (10.6)	35 (33.7)	23 (22.1)	25 (24.0)	10 (9.6)
Lack of contact time with health care professionals (*n* = 106)	2 (1.9)	14 (13.2)	26 (24.5)	39 (36.8)	25 (23.6)
Lack of suitable services (*n* = 106)	1 (0.9)	17 (16.0)	23 (21.7)	36 (34.0)	29 (27.4)
Long waiting times for support services (*n* = 105)	1 (1.0)	13 (12.4)	19 (18.1)	39 (37.1)	33 (31.4)
Stigma around disclosing mental health problems (*n* = 105)	0 (0.0)	14 (13.3)	27 (25.7)	33 (31.4)	31 (29.5)
Stigma around disclosing problems with couple relationship (*n* = 104)	0 (0.0)	10 (9.6)	24 (23.1)	37 (35.6)	33 (31.7)
Barrier to practitioner providing support to couples					
Absence of suitable screening tool (*n* = 97)	20 (20.6)	24 (24.7)	15 (15.5)	19 (19.6)	19 (19.6)
Absence of suitable service to refer couple to (*n* = 99)	6 (6.1)	18 (18.2)	23 (23.2)	31 (31.3)	21 (21.2)
Lack of contact with mothers to identify those with birth trauma (*n* = 97)	30 (30.9)	21 (21.6)	24 (24.7)	12 (12.4)	10 (10.3)
Lack of contact with partners to identify those with birth trauma (*n* = 96)	11 (11.5)	15 (15.6)	26 (27.1)	27 (28.1)	17 (17.7)
Lack of training in this area (*n* = 101)	13 (12.9)	18 (17.8)	25 (24.8)	25 (24.8)	20 (19.8)
Lack of time to spend personally supporting parents (*n* = 99)	8 (8.1)	17 (17.2)	26 (26.3)	23 (23.2)	25 (25.3)
Parents reluctance to accept support (*n* = 98)	16 (16.3)	37 (37.8)	34 (34.7)	9 (9.2)	2 (2.0)
Lack of collaboration with other health care professionals (*n* = 98)	11 (11.2)	25 (25.5)	28 (28.6)	21 (21.4)	13 (13.3)

A strong positive correlation was found between practitioners’ rating of *‘stigma around disclosing mental health problems’* and *‘stigma around disclosing problems with couple relationship’* as barriers to parents accessing support (τ_b_ = 0.73, *p* ≤ 0.00). Exploring barriers to practitioners providing support, a positive correlation was seen between rating of *‘lack of contact with mothers to identify those with birth trauma’* and *‘lack of contact with partners to identify those with birth trauma’* (τ_b_ = 0.63, *p* ≤ 0.00).

### Potential improvements to birth trauma support

**Figure 1.**
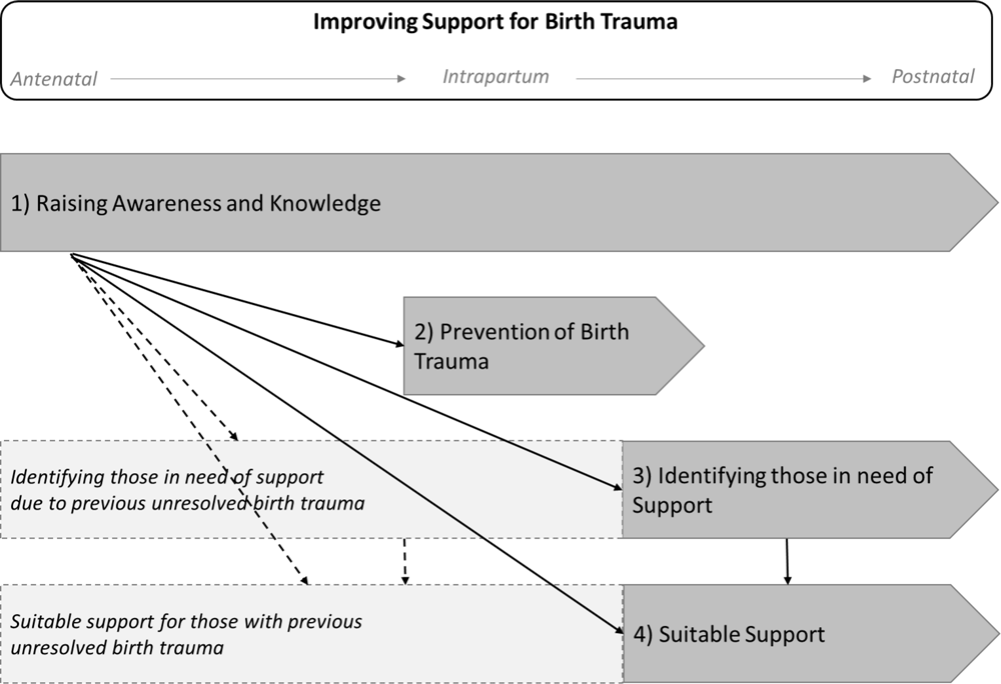
Themes for improving support for parents affected by birth trauma

Four themes emerged from the responses to questions about practitioners’ views on: *‘what are the gaps in support services for couples affected by birth trauma*’ (*n* = 84); *‘what would help couples access suitable support for birth trauma*’ (*n* = 77); and *‘what would help practitioners to support couples affected by birth trauma*’ (*n* = 73). Theme 1) raising awareness and knowledge of birth trauma, 2) prevention of birth trauma 3) identifying those in need of support with birth trauma, and 4) need for suitable birth trauma support. These four themes for potential improvements to birth trauma support are presented with supporting data in Table 6, and Figure 1 proposes how the themes may interact in the perinatal phase.

**Table 6.** Data supporting themes for potential improvements to birth trauma support

Question	What are the gaps in support services for couples affected by birth trauma? (*n* = 84)	What would help couples access suitable support for birth trauma? (*n* = 77)	What would help practitioners to support couple affected by birth trauma? (*n* = 73)
**Theme 1**	‘There is no validation of a traumatic birth ever. So parents go home without any information on support services and are left to suffer alone and do their own research.’ (doula)	‘Information at all stages of contact [with parents], public awareness campaigns’(GP)	‘Wider emphasis on the postnatal period and mental health. Birth trauma is often forgotten!’ (midwife)
**Raising awareness and knowledge of birth trauma**	‘The assumption that healthy Mum and baby must be the only measure of a positive birth, not considering the emotional side of birth.’ (midwife)	‘Greater recognition of trauma in those professionals who see the couples early on…for everybody in the world to never again say “a healthy baby is all that matters”.’ (parent educator)	‘Further training in supporting couples with this specific issue.’ (health visitor)
**Theme 2**	‘In my view, more should be done to avoid the trauma in the first place by addressing the gaps in midwifery training and the lack of woman centered care in birth.’ (lactation consultant)	‘Reducing the point of trauma, ensuring consent, dignity and environment are priorities.’ (midwife)	‘A trauma led care pathway with flagging tool to determine likelihood of trauma and more immediate feedback of couples experiences.’ (midwife)
**Prevention of birth trauma**	‘Securely funded, well signposted local support groups specifically for perinatal issues…thorough physical and mental postnatal check from GP and midwife and health visitor where appropriate, which include questions designed to flag potential ptsd’. (parent educator)	‘All healthcare professionals involved in birth should have training on how to prevent birth trauma.’ (health visitor)	‘Autonomy to discuss in immediate postnatal period without fear of reprimand for causing complaint.’ (midwife)
**Theme 3**	‘Often difficult to see as couple due to partner working or not wanting to admit there is a problem.’ (health visitor)	‘A thorough postnatal screening tool used across the professions for both parents with adequate services to back it up.’ (parent educator)	‘Better communication between the multi-professional teams.’(health visitor)
**Identifying those in need of support with birth trauma**	‘Issues not being picked up or diagnosed quickly enough after birth. Watching and waiting too long sometimes.’ (health visitor)	‘Continuity of care, someone…who had built a relationship of trust so couples would feel able and willing to disclose deeply personal problems.’ (parent educator)	‘Protected time to spend talking about birth experiences with every couple soon after birth.’ (health visitor).
**Theme 4**	‘There’s virtually nothing unless either the person is incredibly debilitated by the trauma and can’t look after their child or the person actively demands help.’ (counsellor).	‘Publicising really clearly what is on offer and destigmatising the support.’ (parent educator).	‘Better joined-up services, clear referral/support pathways, access to written materials or online resources.’ (health visitor)
**Need for suitable birth trauma support**	‘Support available weekends and evenings once father returns to work.’ (health visitor)	‘More services and less waiting lists. Immediate referral after traumatic delivery in hospital.’ (midwife)	‘Easy to refer service with specialist knowledge.’ (family support worker)

Theme 1, raising awareness and knowledge of birth trauma, was highlighted as important for both parents and practitioners. Within this theme was a need to improve information to parents and enhanced training for practitioners in how to support couples with birth trauma. Theme 2 was prevention of birth trauma, which was identified as possible through improvements to intrapartum care to ensure care is respectful and based on informed consent. Likewise, improvements to mental health care could enable secondary prevention of PTSD after birth trauma.

Theme 3 was identifying those in need of support with birth trauma. This was highlighted as possible through practitioners having more time to spend with parents and continuity to build a relationship, particularly with partners. Similarly, increased communication between practitioners was reported as a potential facilitator to recognising parents in need of support, as was having an effective birth trauma assessment tool. Theme 4 was the need for suitable birth trauma support. Practitioners expressed the need for clearer referral pathways to specialist services that can address differing levels of birth trauma distress. Such support needing to be available in a timely way and be accessible by women, partners, and couples.

## Discussion

The findings presented in this paper aimed to examine health care practitioners’ views of the support women, partners, and the couple relationship require when affected by birth trauma, barriers to gaining such support, and potential improvements. Perception of support needs varied according to whether it was for the women, partners, or the couple as a unit. The most offered support was practitioners listening to parents, followed by referral to birth debriefing, and signposting to self-help. Findings highlighted potential barriers to getting birth trauma support along with insight of possible improvements.

Results suggest that practitioners see less demand for birth trauma support from the couple as a unit, or partners, compared to women. Conversely, practitioners highlighted lack of contact with partners as a barrier to providing birth trauma support, and a potential improvement being enhanced services for partners. Further indications that perinatal health services need to consider the needs of partners (Burgess and Goldman, 2018) and engage them in support services (Nystedt and Hildingsson, 2018). Collaborative working in perinatal mental health is associated with reducing barriers to parents accessing support (Smith *et al.*, 2019) and more effective support for families (Myors *et al.*, 2013). The findings of the current study support these findings and suggest improved communication between professionals could improve the identification of couples in need of support. This is pertinent as current variation in birth trauma assessment creates a barrier to effective support through omission of those in need (Delicate *et al.*, 2020).

Despite no conclusive evidence for the routine use of birth debriefing (Bastos *et al.*, 2015), study results are consistent with previous research suggesting it is provided by many post-partum services in the UK (Ayers *et al.*, 2006), with practitioners in the survey perceiving debriefing to be the most effective support for couples affected by birth trauma. Whilst research has shown that some women like and seek out debriefing (Baxter, 2019), its effectiveness as an intervention is questionable and likely to be influenced by its timing and the level of trauma symptoms (Meades *et al.*, 2011).

Similarly, the current study found some practitioners referring to rewind therapy which is not currently an evidenced-based PTSD treatment (NICE, 2018b). However, at the time of this study, rewind therapy was being promoted to NHS practitioners as a birth trauma treatment (Mullan, 2017) and was accredited by the Royal College of Midwives despite no evidence for its effectiveness. This questionable use of rewind therapy for birth trauma may be due to it being available and accredited at a time when health professionals are looking for birth trauma treatments in the absence of any solid evidence about effective interventions for birth trauma. In addition, more intensive, evidenced-based treatments for PTSD may be inappropriate if women or their partners do not have PTSD, as well as being more costly.

Likewise, widespread referral to self-help reported by practitioners in the survey could be due to a lack of suitable services to refer to, or long waiting lists for receiving treatment (Thomson and Garrett, 2019). However, it may serve to reinforce the notion that parents often need to cope on their own with birth trauma (Delicate *et al.*, 2020). Significant differences were found between rates of referral to, and perceived effectiveness for reducing the impact on the couple relationship for: watchful waiting; listening to the couple; signposting to self-help; and referral to third party/charitable organisations. Therefore, some practitioners are referring parents to support they perceived to be inferior, with most practitioners suggesting effective treatment consist of support from multiple sources. This reinforces previous research that more evidenced-based birth trauma interventions (Lapp *et al.*, 2010) and referral pathways are required (NHS England, 2018).

Reported barriers to practitioners supporting parents were in concordance with previous research findings: lack of time to spend with parents (Noonan *et al.*, 2018); limited perinatal mental health training (de Vries *et al.*, 2018); and absence of suitable services to refer parents to (Bayrampour *et al.*, 2018). The correlation between practitioners reporting having the skills and knowledge to support couples and feeling confident in giving support further emphasises the need for increased perinatal mental health training for practitioners (NHS England, 2015; Thomson and Garrett, 2019). There is limited evidence regarding the most effective way to train practitioners, and indeed which practitioners are best placed to support parents with birth trauma.

Developed from the present study results, Figure 1 depicts potential improvements in birth trauma support across the perinatal phase. Increased awareness and knowledge of birth trauma with parents may reduce barriers to parents accessing support (Smith *et al.*, 2019), through easing stigma, improving help seeking behaviours, and developing acceptance of support (Button, 2017). A key element of raising awareness and knowledge of birth trauma in practitioners is through effective professional education and training (de Vries *et al.*, 2018; Thomson and Garrett, 2019). To enable practitioners working with parents to have the time and resources to support parents birth trauma awareness also need to improve at service commissioning and development level (Bauer *et al.*, 2014).

Knowledgeable practitioners and birth trauma aware health care practitioners could help prevent birth trauma. In the antenatal phase prevention can result from assessing and dealing with prior mental health vulnerability and enabling effective birth preparation (Simpson *et al.*, 2018). During intrapartum care, prevention could be enabled by reducing obstetric interventions and facilitating a positive birth experience (Ayers *et al.*, 2016; Dekel *et al.*, 2017). Post-partum, birth trauma aware practitioners and services are needed to help identify those in need of support and assist them in gaining the support they require.

Whilst reassurance and a watch and wait approach is suitable in the early weeks post-partum (NICE, 2018a), the current study suggests that improvements are required to effectively identify women, partners, and couples affected with birth trauma. Consistent with other perinatal mental health problems, once birth trauma is identified, practitioners need clear referral pathways to support capable of addressing varying levels of distress (Noonan *et al.*, 2017). It is recognised that identifying those in need of support, and therefore need for suitable support may be appropriate for some parents antenatally due to prior birth trauma (Greenfield *et al.*, 2019).

### Strengths and limitations

The main limitation of the present study is the use a self-selecting convenience sample which is not representative of the range of post-partum health care practitioners working with parents. Sample size was relatively small and low participant rates from some practitioner groups means their perspective is under-represented in the findings. Study results should be construed as exploratory due to the use of an unvalidated survey, practitioners responding retrospectively based on a range of observations of parents but not clinical diagnoses.

In contrast, the study findings add to a limited body of evidence of health care practitioners’ perspectives of birth trauma. Results illustrate current practice in birth trauma support, practitioners’ proficiency, and insight into how support may be improved. These findings are important for health care practitioners, service providers, commissioners, and researchers engaged in the perinatal phase. Results also have implications for the development of policy and practice to aid the prevention of birth trauma and the provision of effective support services.

## Conclusions

Practitioners indicated that some women, partners, and the couple as a unit require support for birth trauma and identified some barriers to accessing effective support. Suggested improvements include preventing birth being perceived as traumatic in the first instance, raising birth trauma awareness in practitioners and parents, and improving identification of those requiring support. The birth trauma support that is currently being offered is often not evidence based and relies on practitioner’s perceptions of effectiveness and/or availability. It is important that further research is conducted into appropriate birth trauma treatments and that services offer evidence-based support to meet the needs of parents as individuals and the couple as a unit.

## Supplementary material

For supplementary material accompanying this paper visit https://doi.org/10.1017/S1463423620000407. click here to view supplementary material
