# Supplementary material for: Health care practitioners’ views of the support women, partners, and the couple relationship require for birth trauma: current practice and potential improvements
Source: Prim Health Care Res Dev. 2020 Oct 2;21:e40. doi: 10.1017/S1463423620000407 (PMC7576522; doi:10.1017/S1463423620000407)
Supplement: Supplementary file 1 [file S1463423620000407sup001.docx]

Birth trauma and the couple relationship; health care professionals’ views

Q1
​

Title of study: Health care professionals' views of the impact of birth trauma on a couple's relationship and the support required.
 
You are invited to take part in this research study. Before you decide whether you would like to take part it is important that you understand why the research is being done and what it would involve for you. Please read the participant information sheet carefully and discuss it with others if you wish.
 
[Participant information sheet](https://cityunilondon.eu.qualtrics.com/CP/File.php?F=F_1O1p9TdD1sfdSBf)
 
**Do you consent to take part in this study?**

1. I confirm that I have read the participant information sheet. 2. I understand that this study will involve me answering an online questionnaire about my observations of the impact of birth trauma on a couple's relationship. 3. I understand that my participation is voluntary and that I can withdraw from completing the questionnaire at any time.  4. I understand that my participation is anonymous and if I chose to supply an email address I consent to this being used to provide me with a summary of the study results.

- Yes, I agree with the above statements and give my consent to participate in this study
- No, I do not give my consent to participate in this study

Q2 What is your main health care profession?

- Counsellor/Therapist
- Family Support Worker
- General Practitioner
- Health Visitor
- Maternity Care Assistant
- Midwife
- Nurse
- Other - please state ________________________________________________

Q3 How long have you been qualified in this health care profession?

- I am still training
- 0-4 Years
- 5-9 Years
- 10-14 Years
- 15-19 Years
- 20 years +

Q4 What is the first half of the postcode at your main place of work?

________________________________________________________________

Q5 The remainder of questionnaire will focus on your experiences of the impact of birth trauma on parents and specifically the couple's relationship. For this survey, birth trauma is defined as emotionally traumatic childbirth causing ongoing distress. Please base your answers on your experience of working with parents in **the first year after birth**.

Data for questions 6 – 11 not reported in this paper.

Q12 Please rate the following statements regarding your experience of the support couples need for birth trauma

|  | Not at all | A little | A moderate amount | A lot | A great deal |
| --- | --- | --- | --- | --- | --- |
| Mothers want me to support them |  |  |  |  |  |
| Partners want me to support them |  |  |  |  |  |
| Couples approach me together for support |  |  |  |  |  |
| I have the resources to offer support to couples |  |  |  |  |  |
| I have the skills and knowledge required to support couples |  |  |  |  |  |
| I feel confident in supporting couples |  |  |  |  |  |
| I feel that supporting couples is part of my role |  |  |  |  |  |

Data for questions 6 – 11 not reported in this paper.

Data for question 13 not reported in this paper.

Q14
What support do you offer couples affected by birth trauma?
*Select all that apply*

- Watchful waiting
- Listening to the couple
- Self-help information such as books and website
- Charitable/third sector organisations
- Birth listening/debriefing service
- Individual psychotherapy
- Couple psychotherapy
- Couple relationship therapy
- Perinatal mental health service
- Other - please state ________________________________________________

Q15 Which type of support do you feel is most effective in reducing the impact of birth trauma on a couple's relationship?
*Select all that apply*

- Watchful waiting
- Listening to the couple
- Self-help information such as books and website
- Charitable/third sector organisations
- Birth listening/debriefing service
- Individual psychotherapy
- Couple psychotherapy
- Couple relationship therapy
- Perinatal mental health service
- Other - please state ________________________________________________

Q16
Which other organisations do you refer individuals or couples to for support with birth trauma?

*Select all that apply*

|  | Mother | Partner | Couple |
| --- | --- | --- | --- |
| Big White Wall |  |  |  |
| Birth Trauma Association |  |  |  |
| Birth Trauma Chat (Twitter) |  |  |  |
| Children Centres |  |  |  |
| Family Action |  |  |  |
| Homestart |  |  |  |
| Mind |  |  |  |
| NCT |  |  |  |
| OnePlusOne |  |  |  |
| PANDA |  |  |  |
| PIP (Parent Infant Partnership |  |  |  |
| Relate |  |  |  |
| Tavistock Relationships |  |  |  |
| The Relationship Foundation |  |  |  |
| Other - please state |  |  |  |

Q17 In your view what are the gaps in support services for couples affected by birth trauma?

________________________________________________________________

________________________________________________________________

________________________________________________________________

________________________________________________________________

________________________________________________________________

Q18 In your view, which barriers prevent couples accessing support services for birth trauma?

|  | Not at all | A little | A moderate amount | A lot | A great deal |
| --- | --- | --- | --- | --- | --- |
| Concern that requesting support will imply poor parenting |  |  |  |  |  |
| Lack of awareness of birth trauma |  |  |  |  |  |
| Lack of childcare provision to access support service |  |  |  |  |  |
| Lack of contact time with health care professionals |  |  |  |  |  |
| Lack of suitable support services |  |  |  |  |  |
| Long waiting times for support services |  |  |  |  |  |
| Stigma around disclosing mental health problems |  |  |  |  |  |
| Stigma around disclosing problems with couple relationship |  |  |  |  |  |
| Other - please state |  |  |  |  |  |

Q19 What would help couples access suitable support services for birth trauma?

________________________________________________________________

________________________________________________________________

________________________________________________________________

________________________________________________________________

________________________________________________________________

Q20 Which barriers prevent you from providing support to couples with birth trauma?

|  | Not at all | A little | A moderate amount | A lot | A great deal |
| --- | --- | --- | --- | --- | --- |
| Absense of suitable birth trauma screening tool |  |  |  |  |  |
| Absence of suitable services to refer parents to |  |  |  |  |  |
| Lack of contact with mothers to identify those with birth trauma |  |  |  |  |  |
| Lack of contact with partners to identify those with birth trauma |  |  |  |  |  |
| Lack of training in birth trauma |  |  |  |  |  |
| Lack of time to spend personally supporting parents |  |  |  |  |  |
| Parents reluctance to accept support |  |  |  |  |  |
| Lack of collaboration with other health care professionals |  |  |  |  |  |
| Other - please state |  |  |  |  |  |

Q21 What would help you support couples effected by birth trauma?

________________________________________________________________

________________________________________________________________

________________________________________________________________

________________________________________________________________

________________________________________________________________

Data for question 22 not reported in this paper.

Q23 Would you like to receive a summary of the results from this study?

- Yes
- No

Q24 To receive a summary of the results of the survey please email your request to ANON
